# A Change in Work-Family/Life or a Return to Traditional Normative Patterns in Spain? Systematic Review

**DOI:** 10.3389/fsoc.2022.807591

**Published:** 2022-05-31

**Authors:** Almudena Morero-Mínguez, Marta Ortega-Gaspar

**Affiliations:** ^1^Department of Social Work, University of Valladolid, Segovia, Spain; ^2^Department of Constitutional Law and Sociology, University of Málaga, Málaga, Spain

**Keywords:** COVID-19 pandemic effects, work-life/family conflict paradigm, gender inequality, Spain, retraditionalization, family and reconciliation policies, resignification of work/family conflict paradigm

## Abstract

Family policies to reduce conflict in work-life balance and promote gender equality advanced significantly at the legislative level in Spain in the first decades of the twenty-first century. These advances include the 2007 Law for Equality between Men and Women and the extension of paternity leave to 16 weeks in 2020. However, advances in care work and at the professional level have been limited. The COVID-19 pandemic has intensified existing imbalances in family-work responsibilities in general and the ICT gender gap in particular. In crisis situations, women adopt the role of caregivers more easily than men, and women with fewer educational, economic, and job resources are more likely to assume this role, contributing to increasing gender inequalities at work and in the family. COVID-19 has exposed these imbalances, highlighting the need for new narratives and laws that encourage gender equality. Post-COVID-19 scenarios thus present an opportunity for reflection and progress on Spanish family policy. From this perspective, the paradigm of work-family conflict, although interesting, must be examined and resignified. This article proposes to critically resignify the paradigm of work-family conflict based on the new narrative generated by COVID-19. The present analysis suggests a resignification that should involve changing the expectations and practices around work-family balance, based on family diversity, job insecurity, the technological revolution, and new masculinities. It is proposed a prior reflection to clarify definition of the indicators and indexes that enable operationalization of the concept of work-family reconciliation. It is expected that these measures will help to facilitate practical application of reconciliation in areas such as public or/and private organizations, while also enabling international comparative analysis.

## Introduction

Parallel to the increase in dual earner families, significant advances have been made in family policies for work-family reconciliation, primarily through the extension of paternity leaves. These advances have encouraged fathers to take more time off work to care for their children during the first years of their lives (Balsas, 2022). Despite the advances observed in work-family reconciliation policies, the gradual increase in such family policies has not eradicated asymmetries in care work in Spain.

The COVID-19 has meant a reorganization of the public and private spheres, especially affecting care work in families and raising new questions about how the pandemic has affected the organization of the division of labor in the family and thus work-family reconciliation. The research performed seems to indicate that the beginning of the lockdown and subsequently remote work opened new possibilities for renegotiating the distribution of domestic work in couples (Ayuso et al., 2020). It is too early to evaluate the real influence of these changes, as more time and long-term databases are needed to provide accurate information on the situation over time. We can and should, however, reflect on the possible effects of the pandemic on strategies and time scheduling for work-family reconciliation in Spain. It should be taken into account that Spain is a country that experienced a lockdown far stricter than other that is a point that may also explain stronger pressures on women.

Studies of the effects of the pandemic on families' work agree in stressing that families with children have seen an increase in domestic work. This increase has been borne primarily by women, increasing the inequality in domestic work between the sexes, although fathers' involvement in care has also increased (Del Boca et al., 2020; Power, 2020; Seiz, 2021). Studies from a labor economics perspective have shown that the pandemic has had neutral gender effects on the employment of family members with children, primarily in liberal countries (Hupkau and Petrongolo, 2020; Qian and Hu, 2021) and in Italy (Brini et al., 2021). These studies of the work patterns of families with children do not pay sufficient attention, however, to how the pandemic has influenced unpaid^1^ work in the family from the perspective of reconciliation.

One of the questions we ask in this theoretical reflection is whether the pandemic has meant a normative retraditionalization of care work or, on the contrary, is giving rise to new negotiation within households due to the renegotiation of care tasks caused by consolidation of paid work at home. The gender gap that exists in the work world is still present in the world of remote work. A division of roles in the workplace persists, with men less inclined to telework and women more inclined to work remotely due, it seems, to their position within the company. Since working at home has traditionally been seen as a woman's job (the woman was in charge of work in the home), it has been seen as easier for women to combine these tasks (Sullivan, 2019). This reflection requires us to contextualize reconciliation practices properly in both the institutional context in which they occur and the historical tradition that has characterized work-family reconciliation in countries in southern Europe, such as Spain and Italy (Ortega-Gaspar, 2011, 2013; Moreno Mínguez and Crespi, 2017) and in the evolution of social reconciliation policies (Ortega-Gaspar, 2013).

This reflection is performed in light of theories of family change and reconciliation in the context of family policies developed by the Spanish welfare state in recent decades. This exercise in theoretical reflection enables us to formulate the appropriate hypotheses for proposing future research studies on the effects of the pandemic on care work in families and gender equality. Without such prior theoretical reflection, it is difficult to define the proper indicators to measure the scope of the effects and of family change. These post-COVID sociological analyses are absolutely crucial to situating and contextualizing the scope of the pandemic's long-term effects on family behavior and on gender equality. The effectiveness of work-family reconciliation policies that we design from now on will depend on our diagnosis as sociologists, based on the theoretical and empirical research performed. The sociologist's work during and after the pandemic is thus key to developing policies to mitigate the effects of the pandemic and guarantee the individual and family wellbeing that have been decimated by the pandemic.

## The Debate on Work-Family Reconciliation in Pre-COVID Sociological Theory

### The Study of Work-Family Reconciliation in Sociological Theory

The study of reconciliation begins with the social and family changes arising from the increase in middle-class women entering the labor market. Developed countries in particular have seen growing interest in this issue, especially since the 1990s. This fact is important for understanding the dominant approaches to studying reconciliation and for perceiving recent changes. The literature review performed for this study confirms that the first stage of studies on reconciliation focused on gender inequalities (Gerstel and Sarkisian, 2005), inequalities in the division of domestic roles, and labor inequalities deriving primarily from the unequal division of domestic roles (Hochschild and Machung, 1989; Crompton and Lyonette, 2006). Currently, due to changes occurring in societies that are more advanced in issues of labor, technology, and the family, the analytical perspective attempts to show the growing diversity of situations that create obstacles to reconciliation. Researchers' interest in studying precarization and job insecurity has recently increased to determine the real reasons for obstacles to promoting reconciliation, and studies argue that these obstacles especially affect workers in the most disadvantaged social positions. Studying work schedules, the distinctive situations of workers who work at non-standard times (Gerson, 2011; Clawson and Gerstel, 2014; Martín Criado and Prieto, 2015; Cano, 2017), and the trend toward telework with its revolution in the distribution of schedules and tasks (Diane, 2002), this new analytical framework becomes especially interesting in the context of the pandemic and the need for a new definition of spaces and times, while recognizing the new social context of a growing variety of new, increasingly diverse family models (Moreno Mínguez et al., 2017).

Recent social changes reveal the need for studies of reconciliation to adopt a structural perspective^2^ that considers the organizational culture of workplaces and occupational and gender inequalities, as well as the role and influence of different social protection policies (for both families and work), especially those promoting reconciliation (Lim and Misra, 2019). Growing market segmentation, successive economic crises, and the increase in inequalities make it crucial to understand and explain growing complexity around the current need for reconciliation. We must also keep in mind the need for more in-depth study of the perspective of companies and employers.

The very definition of reconciliation has been refined and revised in different times and contexts. The definition of the theoretical concept is highly sensitive to social changes, especially those involving gender issues [some definitions focus on the notion of the need to balance worlds with demands made, others underscore the convergence of the different environments (Junter-Loiseau and Tobler, 1999), and still others call attention to the eternal dilemma of reconciliation, or contradiction (Tobío, 2005; Crompton, 2006; Daly, 2010)].

In Spain, Torns et al. (2002) and Ortega-Gaspar (2013) highlight the strong influence of cultural elements in behavior concerning reconciliation and the values and norms that men and women adopt in a social contract that promotes unequal distribution of work among genders. In the same vein, studies like Moreno Minguez (2011) provide a more holistic and comprehensive definition, viewing work-family reconciliation as a practice that corresponds to the determining subjective and gender-related factors inserted into cultural and institutional frameworks that explain the differences observed among different countries. Social environment and institutional factors such as social and employment policies influence individuals' decisions about reproduction and child rearing (Esping-Andersen, 2000; McDonald, 2001). More specifically, Esping-Andersen (2000) questions the extent to which motherhood is compatible with practicing a profession and observes that the highest fertility rates are found in the countries with the most-developed family policies (especially policies that focus on providing universal free high-quality childcare services). This trend has not been confirmed in the case of Spain in the past decade, where family policies—specifically parental leaves—are highly developed but the gendered division of family work remains unequal (Garrido and Chulía, 2021). The key to this behavioral ambivalence may be found in a crucial issue, that families retain traditional cultural normalization in family practices despite the advances in family policies. That is, the advances in the public sphere reflected in indicators on women's jobs and education are not matched by advances in family work, where inequality is manifest. The interpretive paradigms of work-family reconciliation have found it difficult to incorporate the distinctive characteristics of the Mediterranean family model of reconciliation when interpreting this ambivalence (Moreno-Mínguez et al., 2019). We see proof of this difficulty in studies conducted during the pandemic on liberal countries such as the United Kingdom, which stress that they find no evidence of retraditionalization of gender roles (Galasso and Foucault, 2020; Hupkau and Petrongolo, 2020). The situation is very different in familialist societies like Spain and Italy, two countries that share lower indices of gender equality in terms of women's employment, division of family labor, and redistributive policies to support families. These policies are narrower in scope and less focused than those of other countries (Thévenon, 2011; Daly and Ferragina, 2018; Matteazzi and Scherer, 2020; OECD, 2021).

Caregiving and time are the keystones of reconciliation, as they condition women's job, training, and leisure opportunities, focusing attention on reconciliation with a clear gender bias. The best theoretical approach to reconciliation—by integrating caregiving, gender, and time management—has been the cultural one. From this perspective, most studies of the division of family labor have used a limited definition of the concept, usually focusing on attitudes, beliefs, and values as cultural elements that influence the normalization of individual behavior. Such studies thus examine the extent to which these beliefs and values significantly influence involvement in both paid and unpaid work but neglect to relate these values to the institutional context of family policies and individual factors such as education and the household's perspective.

As to the importance granted to paid work, studies such as Mannheim (1993) warn that women generally tend to be less oriented to employment than men, although more in-depth analysis shows that this orientation occurs primarily among middle-class individuals. In this vein, preference theory (Hakim, 2000) holds that differences between the sexes in paid work stem from choices that different types of women make and do not consider the interrelation of structural and cultural factors when explaining values related to family work and jobs. This is a limited interpretive paradigm unsuited to the characteristics of the Mediterranean model of reconciliation and thus inadequate for explaining the retraditionalizing complexity of the pandemic's effects in Spain. The pandemic has revealed the insufficiency of paradigms of reconciliation to respond to the retraditionalization found in countries in southern Europe. Previous studies as Grünberg and Matei (2020) have proposed the need of a revision of the conflict paradigm within which reconciliation policies are usually designed. The context of the COVID-19 pandemic is crucial to examining the impact of multiple roles on women's WFB. Recent research has shown that women continue to struggle with juggling work and family responsibilities even while they are working from home. This struggle has resulted in increased stress levels and inter-role conflicts as the blurring of borders has intensified (Bahn et al., 2020; Nash and Churchill, 2020). Ultimately, the consensus is that the pandemic has meant an overload of domestic work for women (Del Boca et al., 2020; Kulic et al., 2020; Biroli et al., 2021), even when fathers increase their participation in care (Mangiavacchi et al., 2021; Zamberlan et al., 2021). This retraditionalization has been especially significant in southern European countries, exposing the weakness of reconciliation paradigms in explaining this phenomenon (Brini et al., 2021) and causing among other effects those recognized by the work of Moreno (2002), who clearly emphasizes such imbalances through the term, “the superwomen,” as a symbolic exponent of the Southern European model.

## Reconciliation Policies in Spain As An Example of the Welfare State in Southern Europe

At European level, the need to facilitate reconciliation of personal/family life with work is recognized as a priority for achieving gender equality, increasing women's participation in the labor market, and promoting men's and women's co-responsibility for care. The different member states adopt a series of measures within this framework and the commitment to gender equality. In Spain, from 2016 to 2019, the following policies or action plans were developed and approved by the different governments: the Equal Opportunities Strategic Plan (2014–2016), the Special Plan on the Equality of Women and Men in the Workplace and Against Wage Discrimination (2015–2017=),^3^ the Second Action Plan on Equal Opportunities between Men and Women in the Information Society (2011–2020), the National Strategy for the Eradication of Violence against Women (2013–2016),^4^ the Plan for the Promotion of Rural Women (2015–2018), the Comprehensive Family Support Plan (2015–2017), the Comprehensive Plan to Fight Trafficking of Women and Girls for Purposes of Sexual Exploitation (2015–2018), and the National Protocol against Female Genital Mutilation (FGM), European Commission (2015); MGF (2015). These strategies seek to protect women, promote co-responsibility, establish measures to support fertility, aging, and demographic changes, and eliminate the gender employment gap (20–64). They establish goals of achieving 75% employment for men and women, providing 33% of care for children under the age of 3 and 90% for children 3–6 years of age, and achieving 40% representation of women in positions in which they are underrepresented in companies that quote on the stock exchange (except small and medium-sized enterprises).

As a result of the crisis caused by the pandemic, it has become crucial to detect and determine the difficulties encountered in promoting measures to achieve improved participation of women in the labor market and increase men's involvement in care for dependents. Flexible working arrangements can be an especially powerful tool for retaining women in the workforce. At the same time, the concentration of women in specific employment contracts (e.g., part-time) has a negative effect on earnings, career progression, and pension entitlements, especially if these contracts are extended in the long term [European Institute for Gender Equality (EIGE), 2016]. The status of women's work lives is more likely to be affected by the care needs of others, a probability that can be explained by women's higher uptake of parental leave and position in the labor market—for instance, a higher frequency of part-time work as well as a higher rate of inactivity for women.^5^

As various studies have demonstrated, the structuralist perspective highlights the influence that changes in social policies such as labor regulations and/or aid for care of dependents can have on the phenomenon of reconciliation in redefining paid—and thus unpaid—work. The different social policies that address reconciliation have a philosophical root that can give rise to policies that prioritize developing ways to make life easier for mothers who work outside the home and to reconcile work and family, protecting and promoting children's wellbeing when their parents are working and policies that especially promote support for women who leave the labor market while their children are young (Ortega Gaspar, 2012; Campillo Poza, 2013). The strategies of the public policies developed by each government therefore have different efficacy levels and consequences (Fine-Davis et al., 2004; Fernández Cordón and Soler, 2005; De Villota, 2008). The duration and level of coverage for maternity leaves can also have different types of effects. Further, we must remember that providing incentives for women's employment can benefit their negotiating position in the family but does not ensure better distribution of care (Armijo, 2018). Although Spain has experienced growing presence of women in the job market, it continues to show unbalanced distribution of the time men and women spend on caregiving.

In the context of Europe's general trend to promote full employment, special attention has been paid to reconciliation (Recommendation of the European Council on the gradual assumption of initiatives to enable reconciliation 92/241/CEE). Campillo Poza (2013) observes that the first stage of these policies was driven by a commitment to equal opportunities, which then shifted to development within the framework of the European Employment Policy. Fernández Cordón and Soler (2005) stress that European action on reconciliation has a double origin: great concern for policies to foster gender equality and the growing focus on the aging population and subsequent scarcity of the potentially active population in the medium term. In the countries of southern Europe (like Spain and Italy), the near non-existence of a family policy that can meet these demands (as well as the distinctive structure of their job markets) is a factor influencing the increase in the labor and economic cost of having children, causing demographic problems such as lack of generational replacement and the growing and inevitable process of population aging (Ortega Gaspar, 2012).

Reconciliation policies address different dimensions of this situation, depending on how they focus on policies that provide services; childcare and long-term care services for children, the elderly and/or other dependent relatives; leave arrangements and flexibility (flexible arrangements for working hours); or financial aid. It is recognized that failure to promote work-life balance policies in general, and the lack of childcare facilities in particular, is a major obstacle to women's economic independence. “Reconciliation policies” in general, and provision of childcare facilities in particular, enable both women and men to achieve economic independence. As the OECD (2012) affirms, the greatest pay differences are found in countries where there is little provision of childcare facilities for young children. Among the policies that provide childcare services, authors such as Kamerman (2000) observe that having solid social policies that provide institutional aid for caregiving and children's needs strengthens the labor market and improves women's occupational status. Therefore, the more general these benefits are, the greater the rate of women's participation in the labor market. Availability and accessibility of childcare services differs tremendously throughout Europe (Ortega Gaspar, 2012). Following the data from Plantenga et al. (2005), Spain's policies for provision of childcare have been very limited, especially for children 0–3 years old. Data from 2020 (EU-SILC) show that only 21.6% of children under 3 receive formal care for 30 or more hours per week. Growth in this area has been minimal; the 2010 percentage was around 20%. The percentage is higher for children under 3 who attend childcare for 1–29 h/week, but it remains low compared to other EU member states (23.9%). Spain has not achieved the goal established at either European or national level (i.e., to cover 30% of childcare for children under 3 and 90% for children 3–6 years of age).

According to data from the European Union's Statistics on Income and Living Conditions (SILC-EU), Spain has managed since 2005 to provide full childcare coverage to children ages 4–5 whose parents requested it, and participation in formal caregiving rose from 40% in 2005 to 44% in 2009. The percentage of children ages 3–6 who attended childcare for up to 29 h/week was around 46.6% in 2015 and over 50% (57.7%) in 2020 (EU-SILC, 2021). The figures for children who attend childcare for more than 30 h/week have hardly changed (40.2% in 2020) and continue to be far from the goal of 90%.

In the past 11 years (2009–2020), the percentage of children cared for solely by their parents has remained constant (EU-SILC, 2021). We must pause over this statistic, as it indicates that nearly half of children are cared for by their parents only in a country that has undergone substantial growth in the number of dual earner families and thus requires new measures to contribute to gender equality so that the need to care for the youngest does not limit job opportunities.

For childcare, the two main instruments are leave policies^6^ and early childhood education and care (ECEC) services (Valarino et al., 2018). Spain childcare leaves system is characterized by fully paid leaves that include maternity, paternity, and nursing leaves and unpaid leaves that can be taken for very long period of time (Meil et al., 2017). The maternity leaves of 16 weeks of fully paid if all the requirements are met, if that is not the case, mothers are entitled to 6 weeks of leave at a flat rate. The paternity leave, 16 weeks for the birth of the child. Spain has experienced a big change (in 1980 the paternity leave was of 2 days for the birth of the child according to Law 8/1980, 10 of March, Worker's Statutes; in 2021, Royal Decree-Law 6/2019 of 1 March 2019 allows 16 weeks' leave for childbirth also for fathers). The nursing leave that consists of an option, for either the mother or the father, of two half-hours breaks or one half-hour shorter working day till the ninth month after birth and, in case it is stipulated in collective bargaining agreements or covenanted with the employer, this leave can be used to extend maternity leave by 2 weeks (4 weeks for public officials). Each parent is also entitled to an unpaid parental leave until the child reaches 3 years as well as the right to reduce working hours until the child is 12 (Meil and Escobedo, 2018).

Spain's leave policy since 1996 has been grounded in the European Council directive that requires member states to introduce legislation on parental leaves so that parents can care for their children full-time for the first 3 months. In theory, this is an individual and untransferable right. The directive ensures a guaranteed minimum in all member states, above and beyond which we find a wide range of national regulations with differences among countries as to payment, length, and rights enjoyed. The length of the leave ranges from the first 3 years of the child's life (e.g., in Spain) to only 3 months (Liechtenstein). In some countries, the leave is not paid. In others, it is, and payments vary from a fixed single amount to payment relative to salary. The level of compensation determines which member of the couple takes the leave. The market sector in which the parents work also influences this decision. In Spain, most men who take paternity leave work in the public sector (Ortega Gaspar, 2012). As Meil et al. (2018) and Flaquer and Escobedo (2020) indicate, mothers are generally more likely to access leave, as well as paid parental leaves and the possibility of job flexibility to benefit their children—again, except in the public sector. In Spain at the start of the twenty-first century, the total number of maternity leaves per year was only a third of the total number of children born that year. The percentage of parents who enjoy the leave is thus very low and is only growing slowly. According to Flaquer and Escobedo (2020), the results of various studies show that parental leaves contribute to a decisive change in the development of new parenting patterns.

In 2007, the Spanish parental leave system was reformulated within the framework of the Law for Gender Equality, Organic Law 3/2007, for the Effective Equality of Women and Man (Ley de Igualdad de Género, Ley Orgánica 3/2007, para la Igualdad de Mujeres y Hombres). This reformulation refocused parental leave policies toward fatherhood and a system of reductions in work time and voluntary, reversible, highly flexible work, while also extending these policies to the contingencies of caring for adults (Flaquer and Escobedo, 2020). This is the first time that Spain has granted parents an individualized right financed by the social welfare system. The law extends and provides flexible options for reduction of work time to care for children from one eighth (previously one third) to half of the parent's work time, extending this right until the child turns 8 (or 12 in the public sector, where the cut-off age was previously 6) and to care of a dependent family member (Flaquer and Escobedo, 2020).

In 2017, parental leave was extended first to 4 weeks and, in July 2017, to 5 weeks. In March 2019, the Spanish government ratified Royal Decree-Law 6/2019, of 1 March, on emergency measures to guarantee equal treatment and opportunities for women and men in employment and occupation, and expanded paternity leave to 8 weeks, while committing to lengthen this leave gradually (Flaquer and Escobedo, 2020). In 2020, the parental leave system was further extended to 16 weeks for men. It is too early to understand the full effects of this law, but its implementation augurs a new advance toward equality in division of roles that follows the trajectory of social democratic welfare states. As Flaquer and Escobedo (2020) suggest, fatherhood and motherhood have varied impacts in most countries, often in the opposite direction of job trajectories. Countries with more active and egalitarian reconciliation policies are, however, achieving a positive relationship between employment and parenthood for both men and women.

Another important general change in behavior observed that also affects the Spanish population is the effect of the pandemic on working from home (remote or online work, telework). More specifically, Spain has seen an increasing trend in telework. According to data from the EPA (Active Population Survey, first trimester of 2021), 11.2% of Spain's working population (2,146,100) worked from their homes over half of the days in the trimester, a figure that has nearly tripled in <2 years (from 4.8% in 2019).

Eurostat data (LFS) record even higher percentages, reaching 15.1% in 2020.^7^ In the pre-COVID-19 context, reconciliation policies had not paid attention to the unique conditions of remote workers and their special needs in matters of social protection related to reconciliation. We must promote legislation on telework within the framework of EU countries, as the EU still has no law specifying what regulates telework, although a frame agreement exists that establishes some recommendations. Following the pandemic, a Ruling of the European Economic and Social Committee (EESC) of 25 March, 2021 recognized “the challenges of telework: organization of the work day, private-work life balance and right to disconnect.” Spain was a pioneer in this area, ratifying in 2020 Royal Decree-Law 28/2020 of 22 September, which regulates remote work.

According to the Eurobarometer (2018), slightly over half of Spaniards (52%) have access to agreements for flexible work time (for example, access to change the time they enter or get out of work), and 23% have used this option. These percentages are much lower than those in countries such as Finland or Sweden, where over 80% of the population has access to this option and over 70% have used it. Few countries provide a universal right to flexible work in these matters. In September 2020, Spain ratified a Royal Decree-Law that regulates remote work—a clear effect of the post-pandemic era. In some countries, the legislation focuses on working parents. Companies' involvement continues to be varied and difficult to generalize, as it depends on the idiosyncrasies of each firm.

Spain's public spending on family benefits increased from 2003 to 2007 but represented 1.6% of the GDP in 2007, a figure well below the average of the OECD countries (2.2%). In 2020, Spain was bringing up the rear among countries with the lowest rate of public spending on family benefits (1.19% of the GDP), while the average among OECD countries is 2.11 and Denmark and Luxembourg are at the opposite extreme (investing 3.4 and 3.29 of their GDP, respectively) (OECD, 2021). One consequence of Spain's having the lowest family benefits in Europe is its high rate of child poverty. From a comparative perspective, the global redistributive capability of the Spanish system for households with children is very limited and below that of other European systems, such as those in Germany, France, Britain, and even Italy (Cantó and Sobas, 2020). Cantó and Sobas (2020) calculate that government reform of benefits per child for 2019 represented an increase of 17% in the amount of the benefit (from 24.2 to 28.4 euros a month per child) for low-income households and of 100% for households with very low incomes (from 24.2 to 49 euros per child). This increase raises the number of households receiving the benefit by 5% and spending on the benefit by 66%. The efficacy of this policy in reducing child poverty is limited, however, and has not changed the percentage of minors below the poverty threshold, which only decreased from 31.9 to 31.8% after the reform. It is worth noting that these households are led by women, typically single-parent households and family households at an economic disadvantage—households that feel the restrictive redistributive family policies more acutely and that benefit least from the parental leaves allocated to two-parent families.

As Armijo (2018) argues, from ratification of the 1999 Law of Reconciliation to expansion of parenting leave in 2017, Spain has seen limited development in gender equity with low development highlighting advances in the population of mothers and fathers in the most disadvantaged positions on the labor market. As Fernández Cordón and Soler (2019) argue, reconciliation policies generally continue to make uncovered needs the responsibilities of families, especially needs involving young children and the dependence of very elderly persons. In all welfare societies, the role of the state is important. Yet this role seems to contract in times of crisis, causing care work to revert to families and thus to women. This contraction contributes to the vicious circle perpetuating gender inequality and unbalanced distribution of roles, which in turn prompts a return to the reproduction of traditional behavior models.

## The New Retraditionalization Caused by COVID-19

### Effects of COVID-19 on Work-Family Reconciliation

The pandemic has altered the dynamics of our private life and family relationships. These new dynamics are in the process of redefining work life-family reconciliation, as well as the concept of conflict in reconciliation. Public policies must be attentive to these new dynamics and family structures so that they can define mechanisms for family protection sufficient and suitable to the new circumstances and they should consider the idiosyncrasy and features of the Spanish labor market. In 2020, there exists a difference of more than 11-point percentages in relation to male and female employment rate (71.4 and 60%, respectively); part-time employment and temporary contracts are over-represented among women (male and female part time employment rate 6.2 and 22.5 and male and female temporary contracts represent 18.1 and 22.5, respectively) (Eurostat, 2020).

The patterns of employment in couples with at least one child aged 0–14 is as follow: 43.8% are couples where both partners work full-time; 14.1% one partner full-time, one partner part-time; 28.1% one partner full-time, one partner not working and 5.4% both partner not working (EU-LFS, 2019). In relation to the patterns of employment in couples with children, it is observed interesting changes during last years, specifically in the case of both parent working full time there has been a growth of 8.97% points; and couples where “one partner full-time, one partner part-time” the growth has been of 1.25% points.

Men with children still work more hours than women with children specially when they are very young. The 67.2% of employed men in couples with at least one child aged 0–14 work more than 40 h and the 40.7 of employed women in couples with at least one child aged 0–14 do so (EU-LFS, 2019). About the employment status of single parents, in 2019 the 49.6% work full time and 14.2 work part-time.

The social consequences of COVID-19—such as the stress generated by extended use of technologies in remote work, overload of domestic work added to stressful job responsibilities, and lack of job opportunities—have had a significant impact on the balance of private family life, as well as on the division of family work and the resulting gender relationships. As Rodríguez-Rivero et al. (2020) sustain Covid crisis has demostrated that the great myths of gender inequality are still alive in Spain, and that a crisis of this nature can perpetuate them. Studies performed during the pandemic show that the conditions of most people's life-family reconciliation have worsened and, with this change, their wellbeing and quality of life. The negative effects on women—overloaded with care work during the pandemic—have intensified gender inequality. According to figures on paid care work, 11.5% of global employment includes 381 million care workers, two-thirds of whom are women. All studies performed during the pandemic in different national contexts have demonstrated this negative impact (Power, 2020; Ramakrishnan, 2020; Hipp and Bünning, 2021).

Research conducted by Eurofound (2020) provides evidence that the pandemic has negatively affected the egalitarian distribution of family work between men and women worldwide. Among those surveyed, women reported more difficulties than men in reconciling work and private life, especially when asked about the fatigue they felt doing household tasks after work. This situation was exacerbated for families with preschool-age children. The Eurofound (2020) study concluded that online work and the longer hours this work requires negatively influenced work-family reconciliation. The negative balance was intensified for women with children of preschool age who were dedicating most of their time to care. The greatest differences between men and women with young children were reflected in the statements, “It is hard to concentrate on work because of the family” and “The family prevents me from giving time to my work” (Eurofound, 2020, 22). Up to the time of the pandemic, the childbearing years meant a reduction in paid work time for women and an increase for men (Flaquer and Escobedo, 2020), although both men and women preferred to reduce work time during this initial stage of child rearing (Eurofound the International Labour Office, 2017). The pandemic dashed these expectations, creating a scenario in which remote work extends work hours, eroding the boundaries between time devoted to private and to work life, despite the context of a dominant narrative that remote work has improved working conditions and is having a positive effect on workers (Gálvez et al., 2020; Uresha, xbib2020). One of the most significant problems faced by employees working remotely during the pandemic has been the conflict between work and private life, as the line between work and private life has blurred. Defining the boundaries between work and private life is always a salient issue in the case of remote work, but it is particularly problematic due to the unique circumstances of the pandemic (International Labour Organization, 2020). One of the most significant consequences of the COVID-19 crisis is that it has changed perceptions and expectations and has advanced the view that workers must always be available. This change has had a serious negative impact on the progress made to date on work-family reconciliation (International Labour Organization, 2020).

On the other hand, the pandemic has aggravated the precarity and difficulties of specific fragile family structures, such as single-parent families, in facing the overload of family work, intensifying the conflict in reconciliation, increasing stress, and worsening these families' quality of life relative to that of two-parent families. According to Fundación Adecco (2020), 80% of women who head single-parent families have seen both their job and economic situation and their personal-family life reconciliation worsen with the pandemic in Spain. COVID-19 has contributed to increasing inter- and intra-household inequalities. This dimension received very little attention during the pandemic. The economic and labor disadvantages of single-parent families that existed before the pandemic have increased, and the current crisis is affecting more feminized occupations, as well as families at economic and social disadvantage, such as single-parent families, aggravating the gender gap and the conflict in reconciliation (Wachtler et al., 2020; Reichelt et al., 2021). Including the variable “type of household” in post-COVID-19 studies on reconciliation is thus a crucial part of the approach to the new problem of work-family reconciliation created by the pandemic from a gender perspective.

The COVID-19 crisis has revealed the relevance and importance of families for having a network for basic needs caregiving and the shortcoming of our public policies for guaranteeing effective work-family reconciliation that ensures equal rights for men and women. National context, normative practice, and historical tradition in family policies have altered the effects of COVID-19 on the degree of ambivalence and retraditionalization of familialist practices in work-family reconciliation, exposing the cracks and deficiencies in the advances in family policies and family co-responsibility.

As Meil (2002) suggests, family life has for several decades been undergoing a process of change that affects many dimensions of social life—among them, the social protection system. In fact, social protection systems are constructed on different assumptions about how markets function and how the family provides for individuals' wellbeing. In this way, as Meil and Escobedo (2018) sustain leaves (as part of the social protection offer) can help to redistribute working time and care time throughout the life course.

In Spain, various studies hold that the social protection system has barely adapted to these new family and work situations. The pandemic has made this fact even more visible, as the crisis caused by COVID-19 has helped to reveal the characteristics and new demands of a new social panorama that requires the current protection system to adapt to new and future needs. We need in-depth study of the specific characteristics of this new family and work environment so that the social protection system can adapt to a new family reality (increased diversity of family models, increased dual-earner and single-parent families and one-person households, growth in the rate of dependents per household) and work reality (growing uncertainty, precarity, remote work, etc.).

### The Retraditionalization of Reconciliation in Spain: Interpretive Keys

In the case of Spain, successive crises have revealed the family's capability for resilience in facing situations of their members' vulnerability, most recently the 2008 crisis and the current COVID-19 crisis. More specifically, the COVID-19 crisis has revealed families' rapid reaction to the demands imposed by the pandemic on physical and emotional care of people who live together. The studies performed show that lockdown strengthened familialism-traditionalism of caregiving and thus of work-family reconciliation. On the other hand, surveys conducted by CIS confirm that satisfaction with family life and family relationships remained constant during the pandemic and even increased (CIS, 2021). Yet all this evidence of the strength of the family in terms of caregiving networks and satisfaction contradicts the intensification of asymmetrical reconciliation that the pandemic has generated and the decline of the family as reproductive environment (Garrido and Chulía, 2021; Seiz, 2021). In fact, empirical studies performed in Spain provide solid empirical evidence of this retraditionalization in care work (Farré et al., 2020; Seiz, 2021).

How can we interpret this paradoxical trend that the pandemic has reaffirmed and that does not correspond to the advance of family policies in Spain, such as the advances that have occurred in paternity leaves? A review of the literature on reconciliation practices in Spain shows clear ambivalence in attitudes and behavior after the birth of the first child, and this ambivalence is stronger in women. Studies confirm that couples with egalitarian ideas find it very difficult to put their ideas into practice due to the structural context of a precarious and fragmented job market (low paid-job, need to combine more than one job, low and high skill demand) and the weak support for work-family reconciliation in childcare services. Further, studies have confirmed a strict traditional normative mandate for mothers in contrast to a more relaxed normative mandate for fathers in Spain (González and Jurado Guerrero, 2015; Seiz et al., 2019). This normative pressure does not correspond to egalitarian attitudes in the division of family work in the youngest cohorts (Castro Martín et al., 2018). Further, qualitative studies in Spain have shown that families whose circumstances are favorable to work-family reconciliation tend to develop egalitarian practices after the traditionalization of care practices following the birth of their first child (Seiz et al., 2019).

Family anthropology can provide one explanation for this phenomenon. Spain has been a traditionally familialist country, in which family ties are strengthened internally through family caregiving and solidarity within the family, to the detriment of public ties and solidarity (amoral familialism of Banfield, 1958; Putnam, 1995). Socialization processes that have contributed historically to creating a cultural ethos of traditional attitudes and values around family caregiving and internal family solidarity that have been reproduced culturally and intergenerationally to date. This dynamic of cultural reproduction within families has been weakened, in part by the gender revolution triggered in the cultural context of the second demographic transition. Women's growing individualization and economic and job autonomy are encouraging the generalization of non-traditional normative values around reproduction, family formation, division of family work, and reconciliation. This gradual and ambivalent process toward gender equality, developed in the theory of “multiple equilibriums,” is evolving differently depending on the predominant national context (Esping-Andersen and Billari, 2015). The theory of multiple equilibriums is based on the argument of individualization in the second demographic transition, which has encouraged the silent gender revolution and opened the path to gender equality, based on institutional advances in labor issues and family policies for reconciliation in each national context. According to this theory, each country's family reconciliation strategies respond to the interplay of balancing and rebalancing around the advances in gender equality, family policies, and diversity in family structures and relationships.

The empirical analyses performed in Spain have shown that traditional care practices were strengthened during the pandemic in a context of economic decline in which work-family reconciliation policies contracted, contrary to expectations and to the general discourse during the early days of quarantine. According to Seiz (2021), the lockdown generated conditions appropriate to neutralizing the effects of the culture of “presentism” and the rigidity of work schedules—the main obstacles to achieving gender equality (González and Jurado Guerrero, 2015). Studies have confirmed that remote work encouraged a non-traditional division of paid work, primarily in couples with high occupational status. It does not seem, however, to have achieved similar equality in the division of family labor. According to the findings of Seiz (2021), the gender gap seems to have decreased in paid work but not in unpaid work. Women continue to be more overloaded than men with family work. Studies performed during the harshest period of the pandemic confirm the persistence of traditional gender norms in embracing the traditional role of mother, even among mothers with high education levels assigned to non-normative roles in paid work (Farré et al., 2020; Seiz, 2021). It is still too early to evaluate the real effects of the increase in telework on reconciliation, but we must recognize that remote work does not always lead to greater satisfaction and wellbeing, in fact it seems to be quite complicated to balance telework and care (Moré, 2020). Spending more time at home may actually increase situations of conflict. Many workers feel that teleworking prevents them from disconnecting because their office and home are in the same place. Similarly, doing home and work tasks in the same place increases stress. Finally, remote work does not lead to higher productivity, as there are more possibilities for distraction. Women seem to suffer most from the negative aspects of teleworking in this respect. The European Working Conditions Survey finds that, despite the fact that more men are doing ICT jobs, it is mostly women who telework from home. This phenomenon may be due to the empirical reality that women telework from home more than men because they must reconcile family life and work to a greater extent. Data show significant gender differences in work with ICT, and these are significant factors for the company, the country, the gender role, and family life (Eurofound the International Labour Office, 2017).

These results reveal the persistent influence of traditional gender attitudes, while also exposing the increase in socioeconomic polarization in attitudes toward and practices of work-family reconciliation fostered by COVID-19 in Spain. They result from political management of the pandemic during the lockdown and after, which focused primarily on economic and technical issues and neglected aspects of relational and social life of people in microsocial environments, such as households. Sociological analysis has been nearly absent from political management of the pandemic in nearly all national contexts (Martuccelli, 2021). Sociologists of the family warn in different media that we are not paying sufficient attention to the different impact of the quarantine on men and women in households, an impact that translated into a more severe overload on women and in different types of households. This emergency solution, which recuperated traditions from private life of the past, could have consequences for the future sociability of gender equality.

The idiosyncrasy and specific nature of the situation in Spanish society requires considering the tremendous change that lockdown caused in the life of working parents, who had from 1 day to the next to assume caregiving tasks previously shared with grandparents and the other members of the broad network of informal caregivers. COVID-19 is reinstating a cultural ethos present in the familialism characteristic of Spanish society, in which the role of distributing caregiving tasks again falls entirely to the parents in the full diversity of family models. New expectations for parents'/workers' roles emerge, and this new situation seems to suggest the emergence of different definitions of the reality based on prior job status of parents and workers, education level, and age.

## Conclusions: Future Challenges For Reconciliation Following COVID-19

Situations of crisis like the pandemic have impacted private life, testing the validity of paradigms of family change and reconciliation. The critical review this article performs provides clarity to understand and interpret the dynamics being reproduced in private and family life. In the case of Spain, the pandemic has shown the latent traditional trends in reconciliation through family attitudes and values embedded in the cultural ethos—values and attitudes that do not correspond to those toward paid work, where a more egalitarian ideology predominates. This attitudinal duality, strengthened by the pandemic, has amplified the ambivalence toward reconciliation practices and intrafamily gender inequalities.

This article's critical review of the interpretive paradigms grounding empirical studies of private and family life reconciliation reveals the weaknesses of these interpretive frameworks for understanding the complexity of this phenomenon. The current changes and successive crises show that the achievements concerning reconciliation in the public sphere have not been matched in the private, where overload and gender inequality persist (Goldscheider, 2019). These theoretical paradigms have not provided satisfactory responses to the dilemmas and ambivalences shaping reconciliation in the private sphere. This article's fundamental contribution consists of resignifying the effects of COVID-19 on attitudes and practices of reconciliation between private and family life in Spain in light of the critical review of the main paradigms for work life-family reconciliation.

Contrary to the explanations provided by Billari and Esping Andersen's multiple equilibrium theory (2015), one clear effect of the pandemic on the division of family work seems to be retraditionalization of reconciliation strategies in Spain. This empirical evidence suggests that, far from substantial advances, there seems to be a disconnect between family reconciliation practices and the advances that have occurred in family policies, as for example with paternity leave. On the one hand, a discourse and narrative of dual earners and egalitarian reconciliation is assumed, but at the same time overload of family caregiving on women has intensified during the pandemic. It is as if the pandemic had reactivated the traditional normative values present in the cultural ethos of familialism in caregiving that had begun to weaken before the pandemic.

We could say that we are facing the reproduction of traditional normative subjectivities in the dimension of the family that contradict the opening and implementation of egalitarian attitudes present in the subjectivities and narratives about paid work. This interpretation is consistent with the results obtained in prior studies of job reconciliation in Spain before and during the pandemic. All of the foregoing is reflected in cultural ambivalences that make egalitarian advances more difficult in work-family reconciliation practices (González and Jurado Guerrero, 2015). The pandemic has strengthened and exposed the attitudinal ambivalence that existed previously in symbolic representations of the family in Spain (Moreno-Mínguez et al., 2019; Seiz et al., 2019).

The pandemic has reawakened researchers' interest in studying the phenomenon of reconciliation, as it has shown the persistence of inter- and intrahousehold inequality in Spain, but now from a perspective that integrates private dynamics (subjectivities) with public ones (family policies), thus also integrating individual and structural perspectives (Goldscheider, 2019). Households with more economic and educational resources have negotiated the sharing of tasks under better conditions of equality than households with fewer resources, where concern for the economic dimension and for daily survival is imposed on negotiation of reconciliation, which tends to be resolved with traditional normalization of practices (Seiz, 2021). In the same line of research, so-called “fragile” households, such as single-parent households headed primarily by women, experienced family overload without being able to obtain external aid during lockdown and thus having to attend to the demands of remote work. This situation generated by the pandemic has meant clear retraditionalization of reconciliation, in contrast to two-parent households, where more than one parent was available to attend to the demands of care and attention to children.

Ultimately, this theoretical reflection provides significant theoretical evidence for the scholarly debate on the challenges of reconciliation and gender equality that have arisen during COVID-19, focusing attention on the interpretation of the small body of empirical evidence on traditionalizing dynamics that the pandemic has stimulated in Spain despite the progress that has occurred in family policies. This empirical evidence is presented in the context of interpretive paradigms, helping to define analytic frameworks that enable formulation of the right indicators to approach study of post-pandemic reconciliation and enable development of the appropriate reconciliation policies to reduce the family inequality intensified by the pandemic. The new situation caused by the pandemic leads us to more in-depth development of a holistic theoretical model based on Environment Fit Theory^8^ (Bronfenbrenner, 1979; Voydanoff, 2005) to study the phenomenon of reconciliation, tackling the new social and labor idiosyncrasies that have emerged with the pandemic. This situation is characterized by a context of constant job uncertainty, family diversity, technological revolution, and family changes of crucial interest (questioning the fragility of informal social support provided by older “grandparent” caregivers of the youngest members of society, new masculinities).

This study aims to propose questions in need of further research. The new social scenario requires detailed knowledge of the role that the different family models and public policies play in the perception of gender roles, as well as of the inequalities caused by the reproduction of traditional subjectivities and professional practice in different positions and economic sectors that have amplified job precarity and uncertainty (Fana et al., 2020a,b). This knowledge becomes even more necessary today to promote the development of social policies that address the growing casuistic diversity caused to a great extent by the situation the pandemic has created.

One of the lessons taken from the COVID-19 crisis is that people's caregiving tasks are crucial in all societies at all times, and even more so in times of pandemic. This insight could suggest more concrete recognition in the social imaginary (whether more or less consciously) of idea that caregiving roles deserve a higher position in the hierarchy of values throughout society. If societies before the pandemic revolved around models that extolled economic power, COVID-19 has highlighted the value of “caregiving” and “providing care.” If we add to this the fact that all societies on the planet are moving toward chronic aging, can we expect caregivers' status to improve? If so, we could expect men and women to show more egalitarian distribution of these roles. In this context, it is beneficial to promote any political action that seeks to revalue caregiving work, starting with domestic tasks, we believe that the more these tasks are valued, the more progress will be made toward gender equality. We must therefore create incentives for policies that promote the learning and internalization of values of gender equality in the domestic sphere.

As those who have analyzed contemporary shortcomings in this area in Spain suggest, it is crucial to establish measures that respond to the real problems of the present. As Pastor Seller (2020) suggests, we need measures that enable increased investment to extend coverage and improvement of benefits for dependent children and establish a state plan for reconciliation measures that takes into account the distinctive characteristics of the social system (aid at home, school support programs, services for transportation to school, especially in rural or isolated environments, etc.) to strengthen actions to promote awareness of and education for equal opportunities (education on gender and rights, positive parenting programs, family mediation, simplification of the process for claiming child support) from various areas of the administration. To achieve this goal, it is crucial that there be convergence in the policies promoted by the different administrations to provide a broad, diverse set of family policies in accord with social realities and trends.

## Data Availability Statement

The original contributions presented in the study are included in the article/supplementary material, further inquiries can be directed to the corresponding author.

## Author Contributions

All authors listed have made a substantial, direct, and intellectual contribution to the work and approved it for publication.

## Conflict of Interest

The authors declare that the research was conducted in the absence of any commercial or financial relationships that could be construed as a potential conflict of interest.

## Publisher's Note

All claims expressed in this article are solely those of the authors and do not necessarily represent those of their affiliated organizations, or those of the publisher, the editors and the reviewers. Any product that may be evaluated in this article, or claim that may be made by its manufacturer, is not guaranteed or endorsed by the publisher.

## Footnotes

^1^We refer to unpaid work as the work performed by family, friends and/or neighbors without remuneration.

^2^The structural perspective is the paradigm that understands that social structure factors such as occupation, gender inequality, social policies of the society under study, among other factors, influence the analysis, understanding, and explanation of phenomena such as work-life balance.

^3^In draft form in 2015.

^4^A measure that directly addresses the problem of domestic violence but that indirectly contributes to improving gender equality and thus has indirect effects on reconciliation.

^5^“Inactivity” refers to the percentage of persons classified as neither employed nor unemployed in the labor market.

^6^For more details see Meil et al. (2017).

^7^Percentage of employed adults (15–45 years of age) working from home without distinguishing between those who have and who do not have children.

^8^From the Ecological approach, this theory encompasses the analysis of the work-life balance phenomenon, integrating the characteristics of the family, work, and community environments.
